# Effect of multimodal intervention care on cachexia in patients with advanced cancer compared to conventional management (MIRACLE): an open-label, parallel, randomized, phase 2 trial

**DOI:** 10.1186/s13063-022-06221-z

**Published:** 2022-04-11

**Authors:** Chi Hoon Maeng, Bo-Hyung Kim, Jinmann Chon, Won Sub Kang, Kyounglan Kang, Mihye Woo, Il Ki Hong, Junhee Lee, Kil Yeon Lee

**Affiliations:** 1 Department of Medicine, Division of Medical Oncology-Hematology, Kyung Hee University Hospital, Seoul, South Korea; 2 Department of Clinical Pharmacology and Therapeutics, Kyung Hee University Hospital, Seoul, South Korea; 3 Department of Physical Medicine and Rehabilitation, Kyung Hee University Hospital, Seoul, South Korea; 4 Department of Psychiatry, Kyung Hee University Hospital, Seoul, South Korea; 5 Department of Nutrition, Kyung Hee University Hospital, Seoul, South Korea; 6 Department of Nuclear Medicine, Kyung Hee University Hospital, Seoul, South Korea; 7 Department of Sasang Constitutional Medicine, College of Korean Medicine, Kyung Hee University, Seoul, South Korea; 8 Department of Surgery, Kyung Hee University Hospital, Seoul, South Korea

**Keywords:** Cachexia, Cancer, Chemotherapy, Ibuprofen, Exercise, Omega-3-fatty acid, Complementary and alternative medicine

## Abstract

**Background:**

Cancer cachexia (CC) is a multifactorial process characterized by progressive weight loss, muscle mass, and fat tissue wasting, which adversely affects the quality of life and survival of patients with advanced stages of cancer. CC has a complex and multifactorial pathophysiology, and there is no established standard treatment. Therefore, it is often irreversible and a single treatment modality is unlikely to suppress its progression. We are conducting a randomized trial to investigate the efficacy and safety of a multimodal intervention compared to the best supportive care for patients who received palliative chemotherapy.

**Methods:**

Patients with lung or gastrointestinal cancers undergoing palliative chemotherapy are eligible. Patients are randomized into a multimodal intervention care (MIC) arm versus a conventional palliative care (CPC) arm. MIC includes ibuprofen, omega-3-fatty acid, oral nutritional supplement, weekly physical, psychiatric assessment, nutritional counseling, and complementary and alternative medicine. CPC includes basic nutritional counseling and megestrol acetate as needed (i.e., anorexia ≥ grade 2). All interventions are performed for 12 weeks per subject. The co-primary outcomes are change (kg) in total lean body mass and handgrip strength (kg) from the baseline. A total of 112 patients will be assigned to the two arms (56 in each group).

**Discussion:**

The purpose of this study is to evaluate the effect of MIC in preventing or alleviating CC in patients who underwent palliative chemotherapy. As there is no established single treatment for CC, it is expected that the results of this clinical trial will provide new insights to significantly improve the quality of life of patients with cancer. Considering the complex mechanisms of cachexia, the effect of MIC rather than a single specific drug is more promising. In this study, we did not overly restrict the type of cancer or chemotherapy. Therefore, we attempted to measure the effects of complex interventions while preserving clinical situations. Thus, it is expected that the results of this study can be applied effectively to real-world practice.

**Trial registration:**

This clinical trial was registered in the Clinical Research Information Service (KCT0004967), Korean Clinical Trial Registry on April 27, 2020, and ClinicalTrial.gov (NCT 04907864) on June 1, 2021.

## Administrative information

Note: the numbers in curly brackets in this protocol refer to SPIRIT checklist item numbers. The order of the items has been modified to group similar items (see http://www.equator-network.org/reporting-guidelines/spirit-2013-statement-defining-standard-protocol-items-for-clinical-trials/).

**Table:** 

Title {1}	**Effect of multimodal intervention care on cachexia in patients with advanced cancer compared to conventional management (MIRACLE): An open-label, parallel, randomized, phase 2 trial**
Trial registration {2a and 2b}.	KCT0004967: Clinical Research Information Service NCT 04907864: ClinicalTrial.gov
Protocol version {3}	02-Feb-2021, version 2.5
Funding {4}	This research is supported by the Basic Science Research Program through the National Research Foundation of Korea (NRF) funded by the Ministry of Science, ICT & Future Planning (2017M3A9E4065330)
Author details {5a}	Chi Hoon Maeng^1*^, Bo-Hyung Kim^2*^, Jinmann Chon^3^, Won Sub Kang^4^, Kyounglan Kang^5^, Mihye Woo^5^, Il Ki Hong^6^, Junhee Lee^7^, Kil Yeon Lee^8^ 1 Department of Medicine, Division of Medical Oncology-Hematology, Kyung Hee University Hospital, Seoul, South Korea; 2 Department of Clinical Pharmacology and Therapeutics, Kyung Hee University Hospital, Seoul, South Korea; 3 Department of Physical Medicine and Rehabilitation, Kyung Hee University Hospital, Seoul, South Korea; 4 Department of Psychiatry, Kyung Hee University Hospital, Seoul, South Korea; 5 Department of Nutrition, Kyung Hee University Hospital, Seoul, South Korea; 6 Department of Nuclear Medicine, Kyung Hee University Hospital, Seoul, South Korea; 7 Department of Sasang Constitutional Medicine, College of Korean Medicine, Kyung Hee University, Seoul, South Korea; 8 Department of Surgery, Kyung Hee University Hospital, Seoul, South Korea
Name and contact information for the trial sponsor {5b}	Kil Yeon Lee Tel: +82-10-3228-8261 Email: kilyeonlee@khu.ac.kr
Role of sponsor {5c}	KY Lee, the sponsor and principal investigator, designed the framework for this study, recruited researchers, and reviewed and supervised the written protocol. He can access all data and is responsible for management, analysis, and interpretation of data; writing of the report; and the decision to submit the report for publication. Basic Science Research Program through the National Research Foundation of Korea (NRF) funded by the Ministry of Science, ICT & Future Planning(2017M3A9E4065330) provides all costs, including drugs and researchers’ labor, required for this clinical trial.

## Introduction

### Background and rationale {6a}

Cancer cachexia (CC) is a multi-factorial process characterized by progressive weight loss, muscle mass, and fat tissue wasting, and adversely affects the quality of life and survival of patients with advanced stages of cancer [1]. It is known that 30–50% of patients already experience cachexia at the time of initial diagnosis, depending on the type of tumor [2]. Furthermore, CC affects 50–80% of patients with advanced cancer, most commonly with lung and gastrointestinal cancer, and is the main cause of death in 20–30% of patients with cancer [3–5]. The pathophysiology of CC can be explained by various complex mechanisms, including metabolic alterations in protein and fat, changes in hormonal activity, and tumor-driven inflammation [6]. In addition, cancer treatment itself can cause or worsen cachexia due to chemotherapy-induced anorexia, poor oral intake, and decreased physical activity [7, 8]. Therefore, it is often irreversible, and it is difficult to suppress its progression with any single treatment modality.

Megestrol acetate (MA), which can help maintain appetite and body weight in patients with advanced cancer, has not been proven to be effective in improving quality of life or lean body mass (LBM) [9]. It is known that the weight gain caused by MA is mainly because of edema or an increase in adipose tissue [10]. Furthermore, its use is often limited owing to various adverse events (AEs), such as Cushing syndrome, adrenal insufficiency, or thromboembolic risk [11]. Anamorelin, a selective ghrelin receptor agonist, was studied for CC in patients with non-small cell lung cancer in pivotal phase 3 trials. In that study, 12 weeks of anamorelin administration had a positive effect on LBM but not on handgrip strength compared to placebo [3]. However, the effect was modest, and the drug is not currently approved for CC by the United States Food and Drug Administration (FDA) and European Medicines Agency (EMA) [8]. Non-steroidal anti-inflammatory drugs (NSAIDs) have been studied to reduce systemic inflammation in patients with CC. A meta-analysis revealed that NSAIDs may improve the weight of patients with CC, preserving their quality of life, although the level of evidence was weak [12]. Other pharmacological interventions, such as glucocorticoids, cannabinoids, androgens, and prokinetics have been examined to improve CC, but there is no clear evidence to date.

Of note, CC does not indicate a single static state, but develops as a continuous spectrum through several stages [1]. If the patient is relatively early in the cancer trajectory, the symptoms and signs of cachexia can be alleviated with appropriate chemotherapy [8]. However, in late-stage patients with severe muscle wasting, cachexia is often refractory and unlikely to respond to any intervention [1, 13]. Therefore, it is necessary to develop a multimodal intervention to prevent or alleviate CC at an early stage in patients with advanced cancer receiving chemotherapy, even if weight loss of metabolic changes suggestive of CC has not yet been revealed. We are conducting an open-label, parallel, randomized phase 2 trial to investigate the efficacy and safety of a multimodal intervention including anti-inflammation, omega-3-fatty acids (O3FA), nutritional supplement with counseling, physical exercise, psychiatric intervention, as well as Bojungikki-tang, which mediates immune-modulation and reverses chronic inflammation and wasting conditions as a complementary and alternative medicine (CAM) compared to patients receiving the best supportive care.

## Objectives {7}

We hypothesized that a multimodal intervention comprising anti-inflammation, O3FA, oral nutritional supplement (ONS) with counseling by a nutritionist, physical exercise, psychiatric intervention, and Bojungikki-tang, which mediates immune modulation and reverses both chronic inflammation and wasting conditions as a CAM could prevent the development of CC or improve CC in patients with advanced cancer during chemotherapy compared to in those who received the standard supportive care.

## Trial design {8}

This study is an open-label, parallel, randomized phase 2 trial to determine the superiority of multi-modal intervention care (MIC) to conventional palliative care (CPC) in terms of prevention or alleviation of CC. Patients will be randomly assigned in a 1:1 ratio to either the MIC or CPC arm.

## Methods: participants, interventions, and outcomes

### Study setting {9}

This clinical trial is being conducted at the multidisciplinary departments of the Kyung Hee University Hospital, Korea. The departments of medical oncology, surgery, clinical pharmacology, psychiatry, nutrition, nuclear medicine, physical medicine, and rehabilitation are involved in patient recruitment and treatment intervention. A specialist from the Department of Sasang Constitutional Medicine of Kyung Hee University Korean Medicine Hospital is also involved in treatment intervention for CAM.

### Eligibility criteria {10}

The inclusion criteria are as follows: (1) patients who are diagnosed with recurrent or metastatic solid cancer: gastric, colorectal, pancreatic, biliary tract, and lung; (2) patients receiving first- or second-line palliative chemotherapy; (3) Eastern Cooperative Oncology Group performance status 0–2; (4) patients categorized into normal, precachexia, or cachexia by pre-defined stages of CC (precachexia is defined as weight loss ≤ 5% within the last 6 months, anorexia, and metabolic changes, such as glucose intolerance; cachexia is defined as weight loss > 5% within the last 6 months or body mass index < 20 and weight loss > 2% within the last 6 months [1]); (5) age ≥ 19 years; (6) patients who can comply with treatment interventions in the clinical trial at the discretion of the researcher, for example, those who have no restrictions on simple daily exercise, ONS intake, and use of NSAIDs; and (7) patients with adequate organ function. Laboratory findings conducted within 1 week of the study enrolment should be appropriate for the treatment intervention as well as chemotherapy: total white blood cell ≥ 3000 mm^3^, absolute neutrophil count ≥ 1200 mm^3^, platelet ≥ 100,000 mm^3^, hemoglobin ≥ 8.0 g/dL, aspartate transaminase, and alanine transaminase 3 times or less of the upper limit of the reference range, total bilirubin 2 times or less of the upper limit of the reference range, serum creatinine 1.5 times or less of the upper limit of the reference range or estimated glomerular filtration rate calculated by the Cockcroft-Gault equation > 60 mL/min.

Exclusion criteria are as follows: (1) patients who have a history of cardiovascular disease with or without active treatment: congestive heart failure, poorly controlled hypertension ≥ 160 mmHg of systolic blood pressure or 100 mmHg of diastolic blood pressure despite antihypertensive medication, coronary heart disease; (2) patients with bronchial asthma; (3) patients who are unable to intake food or medication orally or have considerable difficulty in adequate oral intake: bowel obstruction, uncontrolled active inflammatory bowel disease, and short bowel syndrome; (4) patients associated with bleeding risk or peptic ulcer disease or contraindication to taking NSAIDs; (5) patients who have taken MA or progestin analogs within 30 days prior to the study participation; (6) patients who are taking aspirin or NSAIDs continuously for more than 1 week at the time of the study participation; and (7) patients who are taking anticoagulants.

Prespecified medical oncologists will conduct chemotherapy for each participant and prescribe concomitant interventional drugs (NSAIDs, O3FA, and ONS) during the study period. Psychiatric interventions will be performed by a psychiatrist. The exercise program will be established by a physiatrist, and each individual participant’s exercise therapy will be performed by trained, certified physical therapists. Regular nutritional counseling for participants will be provided by two nutritionists. A certified Korean medical doctor will provide herbal medication as CAM. A nuclear medicine specialist will conduct and interpret dual-energy X-ray absorptiometry (DEXA) for each participant’s study outcomes, although he will not be directly involved in specific treatment intervention. Finally, surveys of patient reported outcomes, including quality of life, will be provided and conducted by medical oncologists and Korean medical doctors who will be the physicians in charge of actual medical management for the participants.

### Who will take informed consent? {26a}

Medical oncologists who participate as co-investigators in this study will provide informed consent. Patients treated with palliative chemotherapy are potential subjects for this study. If a patient is judged to be potentially eligible, written informed consent is obtained after giving the patient sufficient time to explain the purpose, methods, significance of the study, and the potential risks and benefits.

The consent form contains the following, but not limited to, in accordance with the regulations of the IRB and HRPP of Kyung Hee University Hospital as well as AAHRPP: (1) contact information of the investigator, (2) consent process as mentioned above, (3) the purpose of this clinical study, (4) methods and study procedures in detail (for example, the duration and the number of visits to the hospital for this clinical study), (5) risks and benefits with information about subject safety, (6) information about the study funding including the amount of research funds, (7) participation expenses, (8) target number of cases enrolled, (9) compensation, (10) information on initiation and cessation of study participation, (11) privacy and confidentiality, and (12) specimen collection.

### Additional consent provisions for collection and use of participant data and biological specimens {26b}

In this study, an additional study for biomarkers is planned. For this analysis, 5 ml of whole blood from each participant will be obtained at the baseline, week 6, and week 13 during the study. These biological specimens will be frozen and stored for 3 years. For future analysis, separate consent from the participants and IRB deliberation are required.

## Interventions

### Explanation for the choice of comparators {6b}

Participants are randomly assigned to either the “Multimodal Interventional Care” (MIC) group or “Conventional Palliative Care” (CPC) group in a 1:1 ratio.

### Intervention description {11a}

#### 1. Multimodal Interventional Care (MIC)

1. Intervention with investigational product (IP)

A participant allocated to the MIC group will receive the following medication as IPs for 12 weeks while the palliative chemotherapy continues as planned: NSAID, O3FA, Bojungikki-tang, and ONS. NSAID is provided orally as ibuprofen 200 mg three times a day. O3FA is provided orally at 1000 mg twice a day. Bojungikki-tang is provided orally as 3.75 g twice a day. ONS is in the form of one sachet containing a 200-mL beverage designed for drinking. Each 200 mL ONS contains 9.8 g of protein, 27 g of carbohydrate, 6 g of fat, and 2 g of fiber. The participants will take it twice a day. All prescribed IPs should be taken daily for 12 weeks, unless a participant is intolerable to them. All IPs should be administered to the participants according to the study protocol. To check compliance with the administration of IP, the remaining IPs that the participant could not take will be retrieved at the next visit and the number of remaining medications will be checked.

2) Intervention with a psychiatrist

**Table:** 

Type	Intervention
Psychotherapy	General psychotherapy
Cognitive behavioral therapy for stress	Week 1: stress screening Week 3: talking about cancer, coping with physical symptoms Week 5: finding and changing thoughts causing stress Week 7: learning coping skills or anger management Week 9: coping with physical changes and potential fears
Cognitive behavioral therapy for sleep	Cognitive behavioral therapy for insomnia
Pharmacotherapy	Performed when necessary according to the researcher’s clinical decision

3) Intervention through exercise by a physiatrist

**Table:** 

Type	Intervention
Warm-up exercise	5–10 min: low intensity (< 40% of VO_2_Max) or moderate intensity
Stretching (ROM exercise)	10 mins
Conditioning (Training) exercise	20–30 min: aerobic exercise (ergometer or treadmill) Resistance exercise (60–80% of 1RM, 1 set 10–15 repetition) Flexibility exercise (upper and lower extremity)
Cool-down exercise	5–10 min: low or moderate intensity: cardiovascular and muscle endurance exercise

*VO_2_Max, maximal oxygen consumption; ROM, range of motion; RM, repetition maximum

4) Others

Participants will receive nutritional counseling four times (baseline, weeks 4, 7, and 13) during the study period by hospital nutrition specialists. Through detailed interviews and counseling, participants can be helped to have their daily meals at home.

#### 2. Conventional palliative care (CPC)

Participants assigned to the CPC group will receive their planned chemotherapy with the best supportive care. No additional IP, psychiatric intervention, or exercise will be provided. However, routine nutritional counseling for all patients who are treated with chemotherapy will be provided twice (baseline and week 13) during the study period. In Korea, every patient receiving chemotherapy can be provided with two nutritional counseling sessions under the reimbursement schedule of the Korean National Health Insurance. In addition, at the clinical discretion of the researcher, MA 625 mg once a day can be administered if the participant shows more than grade 2 anorexia or weight loss based on the Common Terminology Criteria for Adverse Events (CTCAE) version 5.0. MA can also be provided to the participant if the subject is categorized as pre-defined precachexia or cachexia.

#### Criteria for discontinuing or modifying allocated interventions {11b}

Subjects may voluntarily discontinue participation in the study at any time. In addition, the investigator may discontinue the intervention or suspend the subject’s participation in this study at any time according to the following criteria: (1) when a subject requests to discontinue part of the clinical trial treatment during the study period or withdraws consent to participate in this trial, (2) when the investigator decides to drop out of the study because the subject’s continued participation in the study poses a risk that outweighs the potential benefit to the subject, (3) when the subject has taken a drug that is expected to affect the evaluation of the safety and efficacy of the interventions, and (4) violation of inclusion or exclusion criteria.

#### Strategies to improve adherence to interventions {11c}

In this study, especially when assigned to the MIC group, there is a concern that the compliance of the participants might be lowered owing to three kinds of IPs to be taken with multiple and frequent visits to the hospital for exercise intervention. To increase compliance of the participants, trained study coordinators will call the patients before each visit to remind them of the study schedule, and even when they move within the hospital (for example, to the treatment room, laboratory, or exercise therapy room), study coordinators will accompany them directly. To assess drug compliance, the remaining IPs that could not be taken as scheduled will be returned and counted at the next visit.

#### Relevant concomitant care permitted or prohibited during the trial {11d}

MA is not allowed to the participants allocated to the MIC group. There will be no restrictions on chemotherapy or radiation therapy required for patients during clinical trials.

#### Provisions for post-trial care {30}

When an adverse reaction occurs during the conduct of this clinical trial, the investigators will apply utmost effort to recover the side effect by administering appropriate treatment and to minimize the financial and social burden on the patient when hospitalization is required because of an adverse reaction directly due to trial participation. Costs for appropriate treatment will be provided even after the clinical trial is completed. The team has joined the insurance compensation scheme for this purpose.

#### Outcomes {12}

##### Primary outcomes

The co-primary outcomes of this study are change in total LBM (kg) and handgrip strength over 12 weeks of intervention. The change from baseline over 12 weeks is defined as the average of the change (kg) from baseline at week 7 and change from baseline at week 13. We adopted this strategy based on previous literature because we agree with the author’s opinion that averaging changes from baseline over two time points during the study period could be a more conservative approach compared to using only data of baseline and week 13 [3]. Total LBM and handgrip strength are measured by DEXA and a dynamometer, respectively.

*The five elements of specification in reporting primary outcome measures*

**Table:** 

Domain	Total LBM and Handgrip strength
Specific measurement	DEXA for total LBM, Dynamometer for handgrip strength
Specific metric	Change (kg) from baseline
Method of aggregation	Mean or median values
Time-point	Total LBM: Average of the change from baseline at week 7 and change from baseline at week 13 Average of the change from baseline at week 7 and change from baseline at week 13 Hand grip strength: Average of the change from baseline at week 5, change from baseline at week 9 and change from baseline at week 13

The assessment of cachexia should include both physical muscle mass and strength of muscle representing functional aspects [14]. Therefore, we adopted study objectives based on the previous literature [3]. The total lean body mass represents a physical indicator, and the handgrip power represents a qualitative marker as function of the muscle.

##### Secondary outcomes

The secondary outcomes are as follows:

1) Changes (kg) in total body mass, LBM of trunk, LBM of both upper and lower extremities, fat mass

**Table:** 

Domain	LBM of trunk, both upper and lower extremities, fat mass, total body mass
Specific measurement	DEXA
Specific metric	Change (kg) from baseline
Method of aggregation	Mean or median values
Time-point	Changes from baseline at weeks 7 and 13

2) Changes (kg) in handgrip strength measured at time-point different from the primary outcomes

**Table:** 

Domain	Handgrip strength
Specific measurement	Dynamometer for handgrip strength
Specific metric	Change (kg) from baseline
Method of aggregation	Mean or median values
Time-point	Changes from baseline at weeks 5, 9, and 13

3) Changes (kg) in body weight

**Table:** 

Domain	Body weight
Specific measurement	Scale
Specific metric	Change (kg) from baseline
Method of aggregation	Mean or median values
Time-point	Changes from baseline at weeks 5, 9, and 13

4) Changes on the anorexia-cachexia scale

The anorexia-cachexia scale will be assessed using the Functional Assessment of Anorexia/Cachexia Treatment (FAACT) version 4, where higher the score, the better is the outcome with a range of 0–156 [15].

**Table:** 

Domain	Anorexia-cachexia scale
Specific measurement	Functional Assessment of Anorexia/Cachexia Treatment (FAACT) version 4
Specific metric	Change (score) from baseline
Method of aggregation	Mean or median values
Time-point	Changes from baseline at weeks 7 and 13

5) Changes in quality of life

The quality of life will be assessed using the European Organisation for Research and Treatment of Cancer Quality of Life Questionnaire (EORTC QLQ)-C30 version 3 [16]. Questions 1 to 28 use a 4-point scale. The scale scores from 1 to 4: 1 (“Not at all”), 2 (“A little”), 3 (“Quite a bit”), and 4 (“Very much”). Half points are not allowed. The range is 3. For the raw score, fewer points were considered to have a better outcome. The subsequent questions 29 and 30 use a 7-point scale. The scale scores from 1 to 7: 1 (“Very poor”) to 7 (“Excellent”). Half points are not allowed. The range is 6. The raw score needs to be calculated using mean values. Subsequently, a linear transformation is performed to be comparable. More points were considered to have better outcomes. Finally, the rate of toxicity with clinical significance and possible relationship to either study intervention will be obtained based on CTCAE v5.0.

**Table:** 

Domain	Quality of life
Specific measurement	EORTC QLQ-C30 version 3
Specific metric	Change (score) from baseline
Method of aggregation	Mean or median values
Time-point	Changes from baseline at weeks 7 and 13

6) Changes in spleen Qi deficiency questionnaire

The spleen Qi deficiency questionnaire is a validated questionnaire that includes 11 questionnaires [17, 18]. Each item is measured on a 5-point Likert scale, which is as follows: 0 indicates not at all or not applicable; 1 indicates a little applicable; 2 indicates moderately applicable; 3 indicates quite applicable; and 4 indicates extremely applicable. The cut-off value is 43.18 (spleen qi deficiency > 43.18 and nonspleen qi deficiency (≤ 43.18).

**Table:** 

Domain	Spleen Qi deficiency
Specific measurement	Spleen Qi deficiency questionnaire
Specific metric	Change (score) from baseline
Method of aggregation	Mean or median values
Time-point	Changes from baseline at weeks 5, 9, and 13

Primary and secondary outcomes will be measured according to a predefined timeline (Table 1).

**Table 1:** Timeline for outcome measurements

Time schedule	W1	W2	W3	W4	W5	W6	W7	W8	W9	W10	W11	W12	W13
**Primary**													
Total LBM^a^	**√**						**√**						**√**
Handgrip strength	**√**				**√**				**√**				**√**
**Secondary**													
Body weight	**√**				**√**				**√**				**√**
Anorexia-cachexia scale	**√**						**√**						**√**
EORTC QLQ C-30^b^	**√**						**√**						**√**
SQDQ^c^	**√**				**√**				**√**				**√**

^a^Total lean body mass (LBM) and other secondary variables (total body mass, LBM of the trunk, LBM of both upper and lower extremities, and fat mass) will be measured using dual-energy X-ray absorptiometry

^b^European Organisation for Research and Treatment of Cancer quality of life questionnaire C-30

^c^Spleen Qi deficiency pattern questionnaire

#### Participant timeline {13}

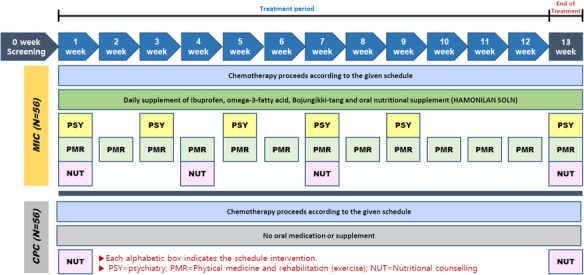



#### Sample size {14}

A sample size of 100 subjects was calculated using statistical program R with the following conditions: two-sample *t* test with a two-sided type I error at 0.5, power of 80%, and 1:1 allocation of MIC versus CPC arm. This sample size was calculated based on handgrip strength which was adopted from the previous study [3], because the standard deviation (SD) of handgrip strength (4.9 kg) was larger than that of LBM. The SD of changes in handgrip strength was assumed to be 4.9 kg in both the treatment arms. Also, the difference of changes in handgrip strength between MIC and CPC arms was assumed to be 2.75 kg. A total sample size of 112 subjects was calculated after considering a 10% dropout rate.

#### Recruitment {15}

Physicians from the Department of Surgery or Medical Oncology are designated as co-investigators to screen all potentially eligible patients, even if they visit our institute for the first time.

### Assignment of interventions: allocation

#### Sequence generation {16a}

A randomization list of all the subjects were obtained from a simple randomization procedure without blocks or stratifications by an independent statistician. This list was generated in a pre-determined computer, which cannot be accessed by other study staffs, to minimize bias when study staffs are aware of random information prior to assignment.

#### Concealment mechanism {16b}

The independent statistician will not be allowed to contact the subjects, and this statistician will generate a randomization list using SAS, version 9.4 (Cary, NC). The randomization list will be prepared in a pre-determined computer with restricted access using a secure password. Eligible subjects will be randomly assigned by this independent statistician.

#### Implementation {16c}

If a patient is determined to enroll by a medical oncologist according to the protocol, the information (screening number, alphabetical initials) for this subject will be provided to the independent statistician. This statistician will assign the enrolled patient to one of the two arms (MIC or CPC) based on the predetermined randomization list. The results of the assignment will be provided to investigators and clinical staff using e-mail, just before the start of week 1.

### Assignment of interventions: blinding

#### Who will be blinded {17a}

This is an open-label study. Blinding is not possible because of the nature of each intervention. However, the person in charge of computed sequencing randomization is independent of the study team and will not know who the participants assigned to each group are. In addition, the data analysts are blinded to treatment allocation.

#### Procedure for unblinding if needed {17b}

This study is not blinded to either the participants or investigators.

### Data collection and management

#### Plans for assessment and collection of outcomes {18a}

Primary and secondary outcomes will be collected according to a predefined timeline (Table 1). The data for outcome variables (total LBM from DEXA, handgrip strength, survey questionnaires for the anorexia-cachexia scale, EORTC quality of life), and the other data for clinical laboratory data or AEs will be primarily collected on the electronic medical reporting system of Kyung Hee University Hospital, which will be reviewed to enter into the electronic data capture tools of REDCap, hosted at Kyung Hee University. Data entry operators should be trained before data collection [19, 20].

#### Plans to promote participant retention and complete follow-up {18b}

All the research teams, including the principal investigator, medical oncologist, and other staff, will have regular meetings to inspect subjects’ compliance and IP accountability at each visit and withdrawal reasons for the subject. In particular, the staff who will contact subjects will communicate their discomfort related to the subject’s compliance, and medical oncologists and dietitians will try to alleviate expected problems for subjects, such as abdominal discomfort and dyspepsia, which can be a hurdle to participate and complete the current study.

#### Data management {19}

Data collection will be performed according to the protocol. These data are stored and managed in the REDCap system [19, 20]. All data will be entered by two pre-determined staff. The quality of data will be regularly assessed by the staff and managers of the REDCap system. The developed electronic CRF in the REDCap system will automatically announce missing values and errors for values outside the predefined range. In addition, the manager will regularly export clinical data from the system to maintain data quality.

#### Confidentiality {27}

All records that could identify the participants will be kept confidential. All documents related to this study, such as the patient’s information or treatment in detail, will be recorded and classified by the subject identification code (subject study number and alphabetical initials) rather than the subject’s name to protect the subject’s privacy and confidentiality. Even when the results of the clinical trial are published, the identity of the participants will be kept confidential, and only the subject’s study number or initials will be recorded when individual data of the subject is to be published or reported.

Clinical trial monitoring personnel and inspectors can view a subject’s medical records to verify the collected information. At this time, the exposed subject’s information will be handled under strict confidentiality, and the investigator will inform the subject of this fact.

Kyung Hee University Hospital will preserve and maintain the security of all data and records related to the conduct of this clinical study. The person responsible for the management and storage of the trial-related documents is the principal investigator, and access is limited to the person in charge of the study. After reporting the study results to the Institutional Review Board (IRB) of Kyung Hee University Hospital after completing the study, informed consent forms and other documents related to the study will be preserved for 3 years in accordance with the Bioethics and Safety Act in Korea.

#### Plans for collection, laboratory evaluation, and storage of biological specimens for genetic or molecular analysis in this trial/future use {33}

All laboratory evaluations are to be performed as a blood or urine test for routine medical purposes (i.e., scheduled chemotherapy). The exceptions are plasma levels of interleukin-2, interleukin-6, and pre-albumin, which are not essential for routine clinical practice. In addition, storage of blood samples for a future study is planned. These samples will be obtained prospectively and frozen three times (screening, week 6, and week 13) with 5 mL of whole blood. These specimens will be abrogated after storage for 3 years.

### Statistical methods

#### Statistical methods for primary and secondary outcomes {20a}

All data will be analyzed using SAS (version 9.4; SAS Institute Inc., Cary, NC) or statistical program R. Primary and secondary outcomes will be described using descriptive statistics (mean, standard deviation and median etc.), and the assumptions of normality will be tested for all the outcome variables to apply appropriate statistical methods: parametric (two-sample *t*-test) or non-parametric (Wilcoxon rank sum test) method. All the outcome variables will be calculated as the change or % change from the baseline measurement, [value at pre-defined schedule (i.e., 5, 7, 9, and 13 weeks) − value at baseline] or [value at pre-defined schedule (i.e., 5, 7, 9, and 13 weeks) − value at baseline]/value at baseline. Two primary outcomes, average of the change in handgrip strength or change in lean body mass, will be analyzed using multiple comparison procedures (Bonferroni, Holm–Bonferroni) to control the family wise error rate. Secondary outcomes will be analyzed as follows: (1) the change or % change from the baseline measurement will be assessed using two-sample *t*-test or Wilcoxon rank sum test at each timepoint [i.e., difference between the values of baseline and 5, 7, 9 or 13 weeks]; (2) all the outcomes will also be analyzed using a statistical model: generalized linear mixed-effects model with baseline outcomes as a covariate, generalized estimating equation, or appropriate nonparametric rank-based methods; all primary and secondary outcomes will be summarized using mean (in the case of parametric tests) or median values (nonparametric tests).

#### Interim analyses {21b}

Interim analysis is not planned for this study.

#### Methods for additional analyses (e.g., subgroup analyses) {20b}

Primary and secondary outcome values will be analyzed using an analysis of covariance (ANCOVA) model, adjusting for each measurement baseline as a covariate. In addition, subgroup analysis will be performed according to categorical variables such as sex or body mass index. In addition, the changes or % changes for the primary or secondary outcomes collected within each time point will be compared using a paired *t*-test or Wilcoxon signed-rank test between the MIC and CPC groups.

#### Methods in analysis to handle protocol non-adherence and any statistical methods to handle missing data {20c}

Data for protocol non-adherence will be dealt with according to the statistical concept of intention-to-treat principle, which will prevent or minimize bias in the statistical analysis. Missing data will be handled based on the International Conference on Harmonization guidelines (ICH E9), entitled Statistical Principles for Clinical Trials. We will analyze the mechanism for the missing data to be handled with suitable methods (e.g., single imputation or multiple imputation, etc.) [21].

#### Plans to give access to the full protocol, participant level-data, and statistical code {31c}

All datasets including the full protocol and law data will be available upon request. Persons who wish to obtain materials can contact the corresponding author through the e-mail indicated on the title page of the published paper.

### Oversight and monitoring

#### Composition of the coordinating center and trial steering committee {5d}

We did not have an independent data and safety monitoring board (DSMB) for this study. However, we believe that our study will ensure adequate quality for the following reasons:

First, our study is regularly monitored by Kyung Hee University Hospital’s Human Research Protection Program (HRPP) system, as well as the auditing group of IRB, to ensure adequate participant registration, and completion of mandatory education for study investigators for clinical trials involvement. Second, monitoring personnel in the study team check whether data is being queried, and provide continuous feedback to the person in charge of data management to prevent errors or omissions each month. Third, because this study is funded by the Ministry of Science, ICT & Future Planning of Korea, study investigators are obliged to submit annual reports on the progress of the research and be audited. Through this, the appropriateness and quality of the study can be guaranteed since these are essential for maintaining the fund. Fourth, in Korea, all clinical research investigators are required to receive a prescribed compulsory education every year for the quality control of clinical studies. Our study team also completed the training for clinical research personnel prescribed by law.

#### Composition of the data monitoring committee, its role and reporting structure {21a}

Based on the standard operating procedure of the IRB of Kyung Hee University Hospital, researchers are required to operate the data monitoring committee if the risk category is 3 (moderate risk without benefit) or higher according to the risk/benefit ratio assessment during the initial review of this study protocol. As this protocol was judged to be category 2 (more than minimal risk with possible benefit), a data monitoring committee was not mandatory.

#### Adverse event reporting and harms {22}

Safety profiles including AEs will be checked and collected at every visit for chemotherapy. All AEs are standardized according to the CTCAE v5.0. Although we did not pre-specify the visit schedule (i.e., every 2 weeks), any harm or adverse events will be checked when the participants visit according to their scheduled chemotherapy sessions. In general, participants will visit and meet the study team every 1–3 weeks for chemotherapy. In the case of dropout participants, safety profiles before dropout should be included in the safety analysis. The causal relationship between adverse events and interventions is determined in accordance with the World Health Organization-Uppsala Monitoring Centre (WHO-UMC) causality assessment [22, 23].

#### Frequency and plans for auditing trial conduct {23}

The Kyung Hee University Hospital’s HRPP system is comprised of the institutional leadership, HRPP manager, audit team, research compliance officer, quality improvement team, institutional review board, office for human research protection, and IRB steering committee (committee of conflict of interest). The IRB works with the HRPP to conduct a study site-visit audit and monitoring. Routine audits are conducted annually between March and November every year. Before conducting the audit, the auditor should carefully review the written plan for the audit target, consent form, and documents submitted to the IRB, and establish detailed audit plans. The auditor assigns an audit number to each audit according to the order of execution of the audit. The audit number shall be prepared as “Year-Audit Type + Audit Order.” At an audit initiation meeting, an auditor explains the purpose, scope, and procedure of the audit to the investigator and receives research-related documents. Based on the predefined “Study Site Visit Checklist for Kyung Hee University,” the auditor shall inspect the data that may affect the reliability and integrity of the trial, such as documents related to consent of the participants (such as consent form), suitability of the target (selected/exclusion criterion), records related to administration/application of medicines/medical devices, and items to be evaluated for primary validity/safety of the research. The auditor may secure a copy of the evidence, if necessary. A closing meeting of the audit shall be attended by an investigator in charge of the research, including a principal investigator, and an auditor who conducted the related audit of the research. The auditor shall conduct an interview with the person in charge of the relevant task at the closing meeting of the audit to check whether the findings are true. In addition, major findings and suggestions are explained verbally, and the audit results should be discussed. The auditor may reflect modifications in the audit results based on the details discussed with the investigator. If there are any lost, incomplete, or incorrect data, an investigator may submit the data to an auditor within 3 days of the audit. The auditor may check the submitted data and reflect the results of the discussion in the audit results. The auditor shall record the list of documents reviewed during the audit and the discussion with the investigators in the “Study Site Visit Checklist for Kyung Hee University.” Of note, all the processes are completely independent of the investigators and the sponsor of this study. The IRB and HRPP of Kyung Hee University Hospital are accredited by the Association for the Accreditation of Human Research Protection Programs, Inc. (AAHRPP).

#### Plans for communicating important protocol amendments to relevant parties (e.g., trial participants, ethical committees) {25}

In the event of alterations, the amended study protocol should be submitted to the IRB for review. A report of amendments made to the previously approved study protocol is requested for submission. In the report, the following items are to be included: (1) description and explanation of amendments made, (2) reason for making amendments, (3) any AEs that occurred under the original research protocol, and (4) expected AEs owing to the amendments. Because this clinical trial is conducted after obtaining approval from the Ministry of Food and Drug Safety of Korea, the amended study protocol and related documents are necessary to confirm whether the Minister of Food and Drug Safety approved the change. However, changes that do not significantly affect the safety of the subjects or the reliability of the test results can be waived.

#### Dissemination plans {31a}

The study results will be published in a peer-reviewed journal. Before that, the abstract of the results can be presented at an academic conference.

## Discussion

When the investigators planned this clinical trial for the first time, it was planned that the MIC group included a total of seven kinds of drug and non-drug interventions, whereas the CPC group included only one routine nutritional counseling. Because the researchers thought that to clearly observe the effect of multimodal experimental interventions, it was necessary to remove other treatments that could potentially cause therapeutic effects in the control group, an issue related to clinical equipoise between the experimental and control groups was raised in the initial IRB deliberation. However, in response to the opinion of the IRB committee, the study team decided to include MA and apply the usual care in real-world practice to the control group. It was decided that MA would be provided when predefined criteria were met for participants allocated to the CPC group. In addition, nutritional counseling will be provided one more time at the end of the study (week 13) to increase the patient’s dietary sand nutritional status.

At the time of writing this manuscript, it has been reported that some participants who have already experienced interventions in the MIC group sometimes complained of abdominal bloating from taking ONS. One pack of ONS has a volume of 200 mL and is to be taken twice a day. In addition to the issues of IP, weekly visits to the hospital for exercise treatment were also difficult in some cases. If there are too many missing parts of the planned interventions, there is a risk of bias in the interpretation of the results after the study is completed. Although this study is ongoing and compliance for IP has not yet been fully investigated, there might be a concern that a number of concomitant interventions may be difficult for patients to implement as intended by a physician in real-world practice. Nevertheless, if multimodal interventions are found to be effective in improving cachexia in this study, it may be possible to provide valuable knowledge to help patients.

## Trial status

The registration date for the first participant was September 15, 2020. At the time of submission, 24 of the 112 subjects were enrolled. The estimated study period is 18 months. The most recent protocol is version 2.5, revised as of February 22, 2021.

## Abbreviations

CC: Cancer cachexia
MA: Megestrol acetate
FDA: United States of America Food and Drug Administration
EMA: European Medicines Agency
NSAID: Non-steroidal anti-inflammatory drugs
O3FA: Omega-3-fatty acid
ONS: Oral nutritional supplement
CAM: Complementary and alternative medicine
MIC: Multi-modal intervention care
CPC: Conventional palliative care
ECOG: Eastern Cooperative Oncology Group
DEXA: Dual-energy X-ray absorptiometry
IP: Investigational product
CTCAE: Common Terminology Criteria for Adverse Events
LBM: Total lean body mass
FAACT: Functional Assessment of Anorexia/Cachexia Treatment
EORTC QLQ: European Organisation for Research and Treatment of Cancer quality of life questionnaire
IRB: The institutional review board
AE: Adverse event
WHO-UMC: World Health Organization-Uppsala Monitoring Centre
HRPP: Human Research Protection Program
AAHRPP: Association for the Accreditation of Human Research Protection Programs, Inc.

## References

[1] Fearon K, Strasser F, Anker SD, Bosaeus I, Bruera E, Fainsinger RL (2011). Definition and classification of cancer cachexia: an international consensus. Lancet Oncol.

[2] von Haehling S, Anker SD (2010). Cachexia as a major underestimated and unmet medical need: facts and numbers. J Cachexia Sarcopenia Muscle.

[3] Temel JS, Abernethy AP, Currow DC, Friend J, Duus EM, Yan Y (2016). Anamorelin in patients with non-small-cell lung cancer and cachexia (ROMANA 1 and ROMANA 2): results from two randomised, double-blind, phase 3 trials. Lancet Oncol.

[4] Dewys WD, Begg C, Lavin PT, Band PR, Bennett JM, Bertino JR (1980). Prognostic effect of weight loss prior to chemotherapy in cancer patients. Eastern Cooperative Oncology Group. Am J Med.

[5] Lim S, Brown JL, Washington TA, Greene NP (2020). Development and progression of cancer cachexia: perspectives from bench to bedside. Sports Med Health Sci.

[6] Argilés JM, Busquets S, Stemmler B, López-Soriano FJ (2014). Cancer cachexia: understanding the molecular basis. Nat Rev Cancer.

[7] Suzuki H, Asakawa A, Amitani H, Nakamura N, Inui A (2013). Cancer cachexia--pathophysiology and management. J Gastroenterol.

[8] Arends J, Strasser F, Gonella S, Solheim TS, Madeddu C, Ravasco P (2021). Cancer cachexia in adult patients: ESMO Clinical Practice Guidelines☆. ESMO Open.

[9] Ruiz Garcia V, López-Briz E, Carbonell Sanchis R, Gonzalvez Perales JL, Bort-Martí S. Megestrol acetate for treatment of anorexia-cachexia syndrome. Cochrane Database Syst Rev. 2013;(Issue 3). Art. No.: CD004310. 10.1002/14651858.CD004310.pub3. Accessed 08 Apr 2022. 10.1002/14651858.CD004310.pub3 PMC6418472 23543530

[10] Loprinzi CL, Schaid DJ, Dose AM, Burnham NL, Jensen MD (1993). Body-composition changes in patients who gain weight while receiving megestrol acetate. J Clin Oncol.

[11] Dev R, Del Fabbro E, Bruera E (2007). Association between megestrol acetate treatment and symptomatic adrenal insufficiency with hypogonadism in male patients with cancer. Cancer..

[12] Solheim TS, Fearon KC, Blum D, Kaasa S (2013). Non-steroidal anti-inflammatory treatment in cancer cachexia: a systematic literature review. Acta Oncologica (Stockholm, Sweden).

[13] Aapro M, Arends J, Bozzetti F, Fearon K, Grunberg SM, Herrstedt J (2014). Early recognition of malnutrition and cachexia in the cancer patient: a position paper of a European School of Oncology Task Force. Ann Oncol.

[14] Solheim TS, Laird BJA, Balstad TR, Bye A, Stene G, Baracos V, Strasser F, Griffiths G, Maddocks M, Fallon M (2018). Cancer cachexia: rationale for the MENAC (Multimodal-Exercise, Nutrition and Anti-inflammatory medication for Cachexia) trial. BMJ Support Palliat Care.

[15] Ribaudo JM, Cella D, Hahn EA, Lloyd SR, Tchekmedyian NS, Von Roenn J (2000). Re-validation and shortening of the Functional Assessment of Anorexia/Cachexia Therapy (FAACT) questionnaire. Qual Life Res.

[16] Fayers P, Bottomley A (2002). Quality of life research within the EORTC-the EORTC QLQ-C30. European Organisation for Research and Treatment of Cancer. Eur J Cancer (Oxford, England: 1990).

[17] Kim J, Kim H, Kim KH (2017). Effects of Bu-Zhong-Yi-Qi-Tang for the treatment of functional dyspepsia: a feasibility study protocol. Integr Med Res.

[18] Kim J, Park J-W, Ko S-J, Jeon S-H, Kim J-W, Yeo I (2017). Effects of a herbal medicine, Yukgunja-Tang, on functional dyspepsia patients classified by 3-dimensional facial measurement: a study protocol for placebo-controlled, double-blind, randomized trial. Evid Based Complement Alternat Med.

[19] Harris PA, Taylor R, Thielke R, Payne J, Gonzalez N, Conde JG (2009). Research electronic data capture (REDCap)--a metadata-driven methodology and workflow process for providing translational research informatics support. J Biomed Inform.

[20] Harris PA, Taylor R, Minor BL, Elliott V, Fernandez M, O'Neal L (2019). The REDCap consortium: building an international community of software platform partners. J Biomed Inform.

[21] Rubin DB (1976). Inference and missing data. Biometrika..

[22] Edwards IR, Aronson JK (2000). Adverse drug reactions: definitions, diagnosis, and management. Lancet.

[23] Nebeker JR, Barach P, Samore MH (2004). Clarifying adverse drug events: a clinician's guide to terminology, documentation, and reporting. Ann Intern Med.

